# Polyphenol Composition, Antioxidant Activity and Cytotoxicity of Seeds from Two Underexploited Wild *Licania* Species: *L. rigida* and *L. tomentosa*

**DOI:** 10.3390/molecules21121755

**Published:** 2016-12-21

**Authors:** Igor Parra Pessoa, José Joaquim Lopes Neto, Thiago Silva de Almeida, Davi Felipe Farias, Leonardo Rogério Vieira, Jackeline Lima de Medeiros, Aline Augusti Boligon, Ad Peijnenburg, Ivan Castelar, Ana Fontenele Urano Carvalho

**Affiliations:** 1 Department of Biochemistry and Molecular Biology, Federal University of Ceará, 60020-181 Fortaleza, CE, Brazil; igorppessoa@gmail.com (I.P.P.); lopes.joaquimm@hotmail.com (J.J.L.N.); thiago.urca@gmail.com (T.S.d.A.); leorv2005@hotmail.com (L.R.V.); jackelinemedeiros@hotmail.com (J.L.d.M.); 2 Department of Molecular Biology, Federal University of Paraíba, 58051-900 João Pessoa, PB, Brazil; davi@dbm.ufpb.br; 3 Health Sciences Center, Federal University of Santa Maria, 97105-900 Santa Maria, RS, Brazil; alineboligon@hotmail.com; 4 RIKILT, Wageningen University and Research, P.O. Box 230, 6700 AE Wageningen, The Netherlands; ad.peijnenburg@wur.nl; 5 Department of Finance, Federal University of Ceará, 60020-181 Fortaleza, CE, Brazil; lume1250@yahoo.com.br

**Keywords:** MCF-7, Caco-2, DPPH, TBARS, phenolic compound, cytotoxicity

## Abstract

Studies have shown the benefit of antioxidants in the prevention or treatment of human diseases and promoted a growing interest in new sources of plant antioxidants for pharmacological use. This study aimed to add value to two underexploited wild plant species (*Licania rigida)* and *L. tomentosa*) from Brazilian flora. Thus, the phenolic compounds profile of their seed ethanol extract and derived fractions were elucidated by HPLC, the antioxidant capacity was assessed by in vitro chemical tests and the cytotoxicity determined using the human carcinoma cell lines MCF-7 and Caco-2. Eleven phenolic compounds were identified in the extracts of each species. The extracts and fractions showed excellent antioxidant activity in the DPPH assay (SC_50_, ranging from 9.15 to 248.8 µg/mL). The aqueous fraction of *L. rigida* seeds was most effective in preventing lipid peroxidation under basal conditions (IC_50_ 60.80 µg/mL) whereas, in the presence of stress inducer, the methanolic fraction of *L. tomentosa* performed best (IC_50_ 8.55 µg/mL). None of the samples showed iron chelating capacity. Ethanolic seed extracts of both species did not reveal any cytotoxicity against MCF-7 and Caco-2 cells. Both plant species showed a promising phenolic profile with potent antioxidant capacity and deserve attention to be sustainably explored.

## 1. Introduction

Brazil is the most megadiverse country on Earth and is the subject of significant global interest and debate regarding deforestation and environmental protection. However, much of the potential of its plant species is still unknown. One strategy to contribute to the conservation of valuable species for solving human health problems is to provide knowledge to awaken public actions aiming to protect this arsenal of plant species, which, besides their role in the natural ecosystems, can also contribute to prevent and treat chronic diseases. Epidemiological studies have shown that a regular intake of fruits and vegetables is associated with low risk of several chronic diseases including cancer, cardiovascular disease and aging in general, their polyphenols content and respective antioxidant activity apparently being responsible for that [1,2,3]. Therefore, there is a growing interest in the discovery of new sources of natural antioxidants in plants for the pharmacological and food/nutraceutical industries [4,5].

The Chrysobalanaceae is a family of trees, shrubs and flowering plants consisting of 17 genera and about 450 species distributed in tropical and subtropical regions worldwide. *Licania* is the most representative genus, which includes the species *L. rigida* Benth and *L. tomentosa* Benth [6,7]. These two *Licania* species are widely spread in Brazil, but more commonly found in the Brazilian Northeast region. In folk medicine, both species have numerous therapeutic attributes. *L. rigida* fruits are used against diabetes with proved hypoglycemic activity and diuretic effects [8]. Seed extracts of *L. tomentosa* have been demonstrated to exert inhibitory activity against the herpes simplex virus, and extracts of its leaves and fruits have been shown to have anti-cancer properties against leukemia cell strains [9,10]. As for the chemical composition, these two plant species have been described to contain flavonoids, tannins and steroids, as well as triterpenoids of the oleanane, ursane and lupane groups [11,12].

A previous study by our group identified promising antibacterial, anticholinesterase and antioxidant activities in ethanolic extracts of seeds from *L. tomentosa* and *L. rigida*, highlighting a biological potential for these species [13]. Thus, the aim of the present study was to characterize and quantify the phenolic compounds of *L. tomentosa* and *L. rigida* seed extracts and their respective fractions, to estimate their antioxidant capacity by different methods and to determine their possible cytotoxicity towards two cancer cell lines The goal for that is to increase attention and investments on wild plants, leveraging the results of scientific investigation, enhancing the link between in situ conservation strategies and sustainable use of plant diversity.

## 2. Results and Discussion

### 2.1. Chemical Characterization

The qualitative phytochemical analysis of the seed ethanolic extracts from *L. tomentosa* (LtEE) and *L. rigida* (LrEE) showed similar overall profile and revealed tannins, flavonoids and saponin classes. The data are presented in Table 1.

The phytochemical analysis can be a first step to guide the subsequent tests, but the assays used for this analysis do not provide much information about the quantity or the proportion of the constituent compounds. For this purpose, we ran three further analyses—determinations of total phenolics, tannins and total flavonoids, as shown in Table 2—which, in turn, confirmed some of the results of the previous analysis and gave us a more tangible perspective on the amount of each type of phenolic compound. In general, *L. rigida* was shown to be richer in tannins than *L. tomentosa*, the aqueous fraction of both species containing the highest levels (0.294 and 0.182 tannic acid equivalent in mg per g of the sample, respectively).The tannin content of the ethyl acetate fractions from both plants was below the detection limit of the method. The total phenolic contents of extracts and fractions of both *Licania* seeds ranged from 96.95 to 206.98 mg of gallic acid equivalent/g of extract which are much higher than those described for some fruits, such as apple (1.91 mg/g), watermelon (1 mg/g), grape (0.93 mg/g), orange (1.95 mg/g) and kiwifruit (3.18 mg/g) [14]. Similarly, the total flavonoids content of LtEAF (90.61 mg of quercetin/g of extract) showed values much higher than those described to apple (0.45 mg/g), mangosteen (0.41 mg/g), pear (0.32 mg/g) and tomato fruits (0.38 mg/g).

**Figure 1:**
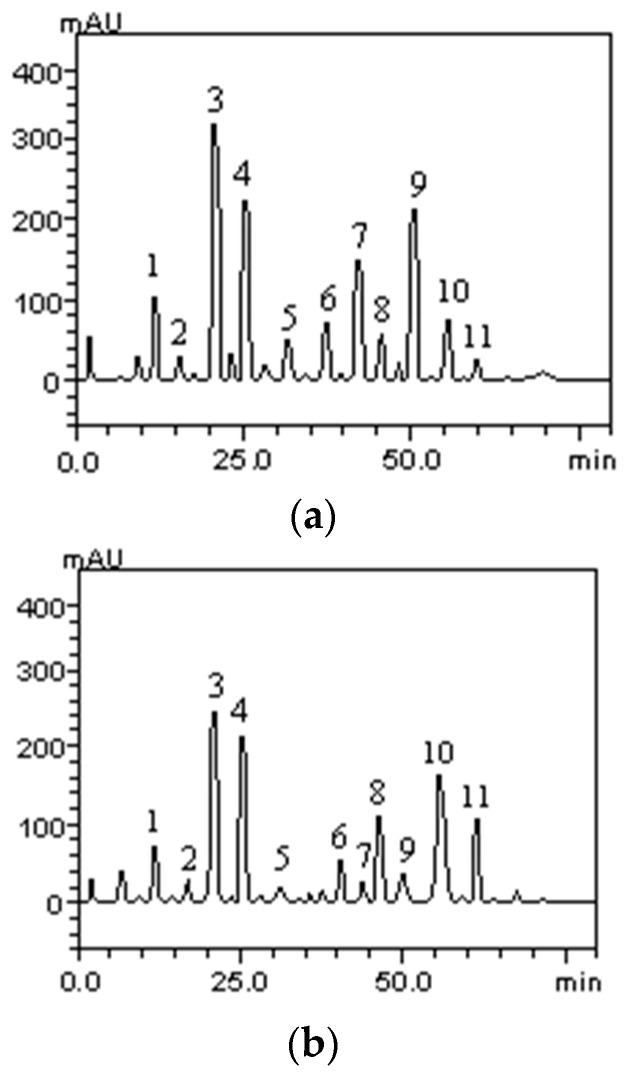
High performance liquid chromatography phenolics and flavonoids profile of *L. tomentosa* ethanolic extract (**a**) and *L. rigida* ethanolic extract (**b**). Gallic acid (peak 1), catechin (peak 2), chlorogenic acid (peak 3), caffeic acid (peak 4), epicatechin (peak 5), ellagic acid (peak 6), rutin (peak 7), quercitrin (peak 8), quercetin (peak 9), kaempferol (peak 10) and kaempferol glycoside (peak 11).

High Performance Liquid Chromatography (HPLC) analysis of the seed ethanolic extracts and their respective fractions indicated that the fractionation procedure used did not generate fractions with different compositions. Two representative chromatograms are shown in Figure 1.

Despite the similar chromatographic profile of the fractions, the constituents were present at different concentrations. For example, in the ethyl acetate fraction of both plant species all the identified phenolic acids and flavonoid compounds were present in much higher concentrations than in the other samples, as described in Table 3 and Table 4. In *L. tomentosa*, chlorogenic acid, rutin and quercetin were the compounds with the highest concentrations. The last two are widely known to be strong antioxidant substances. On the other hand, *L. rigida* showed as main compounds in all the fractions, except for LrEAF, chlorogenic acid, kaempferol and caffeic acid. In this instance, only the latter has been scientifically recognized as having remarkable antioxidant potential [15,16,17,18]. There are reports of other plants of the same genus, such as Licania licaniaeflora, which has been described as having strong antioxidant activity due to the presence of quercetin derivatives whereas for the kaempferol derivatives the opposite pattern is observed [19]. As a matter of fact, the flavonoids quercetin and kaempferol have been used as chemotaxonomic markers in the Chrysobalanaceae family and it has been noted that they are of wide-spread occurrence in the genus *Licania* [20].

### 2.2. Total Antioxidant Capacity

In general, the results showed a wide range of values for antioxidant activities when considering both methods (DPPH and TBARS, the latter with or without iron stress), as described in Table 5. In addition, the LrEAF had an outstanding performance in the DPPH test with values similar to those of ascorbic acid (SC_50_ 9.15 and 10.17 µg/mL, respectively). It is noteworthy that some of these samples could inhibit lipid peroxidation as well as scavenge directly free radicals. If the results were analyzed by considering only the TBARS assay, it could be said that LrAF and LtMF had the best results, without and with iron, respectively (IC_50_ 60.80 and 8.55 µg/mL). As a matter of fact, is the result shown by LrAF is remarkable considering that in most studies, water is rarely used as solvent for extraction or fractionation. Focusing on the DPPH results, the LtEE, LtMF, LtAF, LrEAF and LrAF showed excellent results (SC_50_ ranging from 9.15 to 42.68 µg/mL) compared to ascorbic acid (SC_50_ = 10.17 µg/mL) and the SC_50_ observed for these samples were much lower than that observed in leaves of *L. tomentosa* described elsewhere (SC_50_ 73.33 µg/mL) [21]. The “total antioxidant capacity” (TAC) of a particular sample cannot be accurately measured by any isolated assay because of the chemical complexity of the antioxidant compounds. For instance, the methodologies should be able to evaluate both lipophilic and hydrophilic nature of the phenolic compounds, and distinguish hydrogen atom transfer, electron transfer, as well as transition metal chelation.

The antioxidant activity of these high phenolic-content samples (LtEAF and LrEAF) was disappointing. The former had a bad performance in boths tests whereas the latter had a bad result only in the DPPH assay. This strongly contradicts the literature that normally states that phenolic compounds correlate well with antioxidant activity [22]. It is, however, possible that ethyl acetate solvent must have extracted other compounds that could act antagonistically on antioxidant activity [23]. It is also important to stress that some phenolic antioxidants can auto-oxidize and behave like prooxidants under certain circumstances. Instead of breaking the free radical chain reaction, the phenoxy radical may also interact with oxygen and produce quinones and superoxide anion ($O_{2}^{-}$) [24]. Simple phenolics which are easily oxidized, such as quercetin and gallic acid, possess prooxidant activity [25]. These arguments could partially explain why the samples with high concentration of simple phenolic compounds had poor antioxidant activity.

In the present study, the results on antioxidant activities of the different samples were crossed with the phenolic compounds composition data, so that a correlation could be established. The antioxidant activity measured by the DPPH correlated very well with quercetin (r = 0.788) and quercitrin (r = 0.744). As for the results of TBARS assay without iron-induced stress, catechin (r = 0.690) and quercetin (r = 0.514) presented the best correlations. In general, flavonoids showed better correlation values than the phenolic acids in the results of these two assays. The data are presented in Table 6.

In fact, the flavonoids are recognized for exceptional antioxidant activity [26], and quercetin is very frequently chosen as a positive control in antioxidant assays [27]. Catechin has an analogous chemical structure, but it lacks the oxo group in the carbon 4 and a double bond in the carbons 2 and 3, which confers a less potent antioxidant activity when used alone [28]. When the TBARS was performed with iron-induced stress, a different outcome was observed: the highest correlation values were those of chlorogenic acid (r = 0.465) and rutin (r = 0.346), a flavonoid derived from quercetin. Phenolic acids showed a low positive correlation in this assay (r = 0.246) and flavonoids no correlation at all. In the case of phenolic acids, the interaction with the egg phospholipids and the ability to form stable complexes with iron may have been more important than the mere ability to neutralize free radicals. For the iron chelating property, it is of utmost importance to have one or both two groups: catechol and galloyl. In addition, to give greater protection to the phospholipids, polyphenols must have hydrophilic groups in their chemical structure, such as hydroxyls and carbohydrates that can interact with the polar part of the micelles formed by them [29]. These characteristics are presented by rutin and chlorogenic acid chemical structure [30].

### 2.3. Chelating Capacity

**Figure 2:**
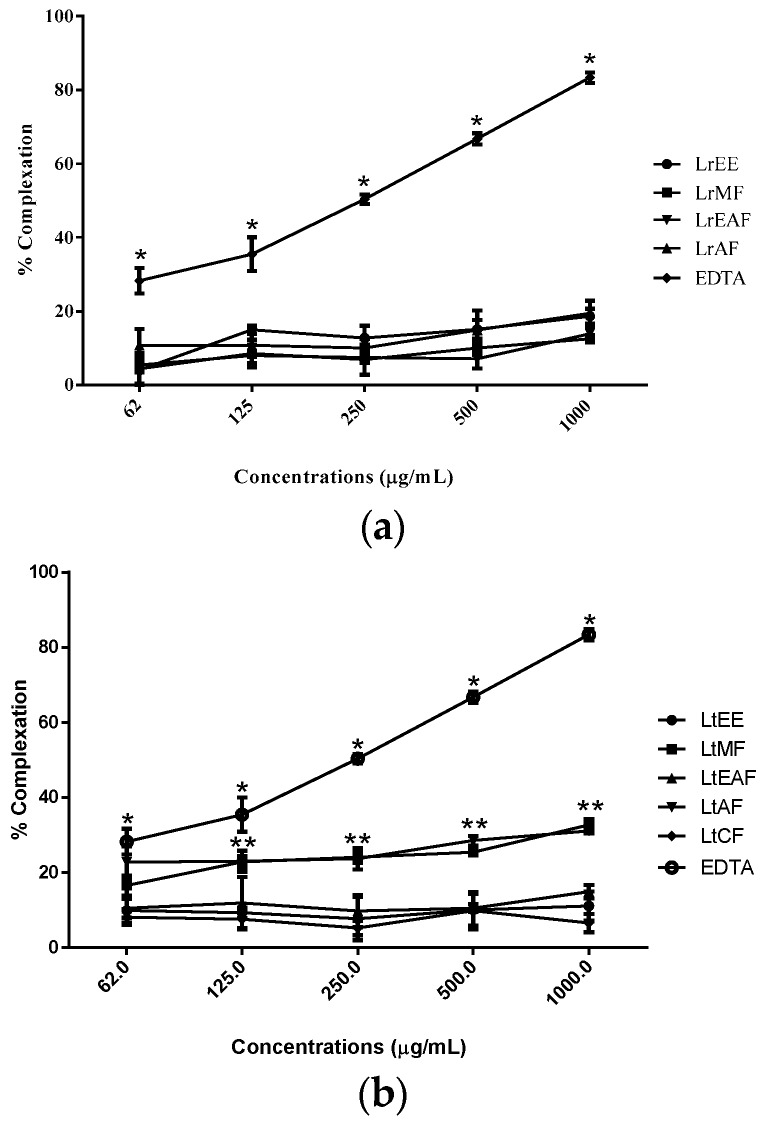
Iron chelating activity of *Licania rigida* (**a**) and *L. tomentosa* (**b**) seeds ethanolic extract and their fractions based on *o*-phenantroline method. Absorbance was recorded at 510 nm and percent complexation was calculated with respect to control (determined in the absence of samples and EDTA). LrEE = *L. rigida* ethanolic extract; LrMF = *L. rigida* methanolic fraction; LrEAF = *L. rigida* ethyl acetate fraction; LrAF = *L. rigida* aqueous fraction LtEE = *L. tomentosa* ethanolic extract; LtMF = *L. tomentosa* methanolic fraction; LtEAF = *L. tomentosa* ethyl acetate fraction; LtAF = *L. tomentosa* aqueous fraction; LtCF = *L. tomentosa* chloroform fraction; EDTA = Ethylenediamine tetraacetic acid. * Significant difference between EDTA control and all the samples in A and B (*p* < 0.05). ** Significant difference between LtMF/LtAF and other samples in B (*p* < 0.05).

As for the *o*-phenanthroline assay, no sample was able to chelate free iron as the positive control EDTA, at least when tested in vitro (Figure 2). However, LtMF and LtAF showed chelating activity higher than the other fractions of *L. tomentosa*. This result is not necessarily negative in what concerns antioxidant potential since chelating capacity is just one among many mechanisms of action to help prevent oxidative stress in living beings [31,32].

In fact, many studies with plant extracts have shown that beyond the capacity to neutralize free radicals, plant compounds show also the ability to chelate transition metals. Indeed, the intracellular redox state is related to the iron (or copper) redox couple and the maintenance of its levels is under strict physiological control [33]. It has been inferred that iron regulation guarantees no free iron is present in the cell. Nevertheless, under stress circumstances, iron levels may surpass the capacity of transferrin to bind it. The released Fe(II) can participate in the Fenton reaction, generating hydroxyl radical [Fe(II) + H_2_O_2_ → Fe(III) + ^•^OH + OH^−^], which is extremely reactive and might impair various cellular components [34]. Thus, it is useful to measure the iron chelating capacity of a sample. The results for this test helped us to abandon the hypothesis that lipid peroxidation protection was only due to the ability to trap iron, turning it less available for the Fenton reaction (Figure 2). It is more plausible to believe that there is more than one mechanism involved in the lipid peroxidation inhibition. Antioxidants are compounds that can delay autoxidation by inhibiting formation or interrupting propagation of free radicals in different ways: (1) scavenging species that initiate peroxidation; (2) chelating metal ions so that they are unable to generate reactive species or decompose lipid peroxides; (3) quenching O^2−^ preventing formation of peroxides; (4) breaking the autoxidative chain reaction; and/or (5) reducing localized O_2_ concentrations [35].

### 2.4. Cytotoxicity to Cancer Cell Lines

**Figure 3:**
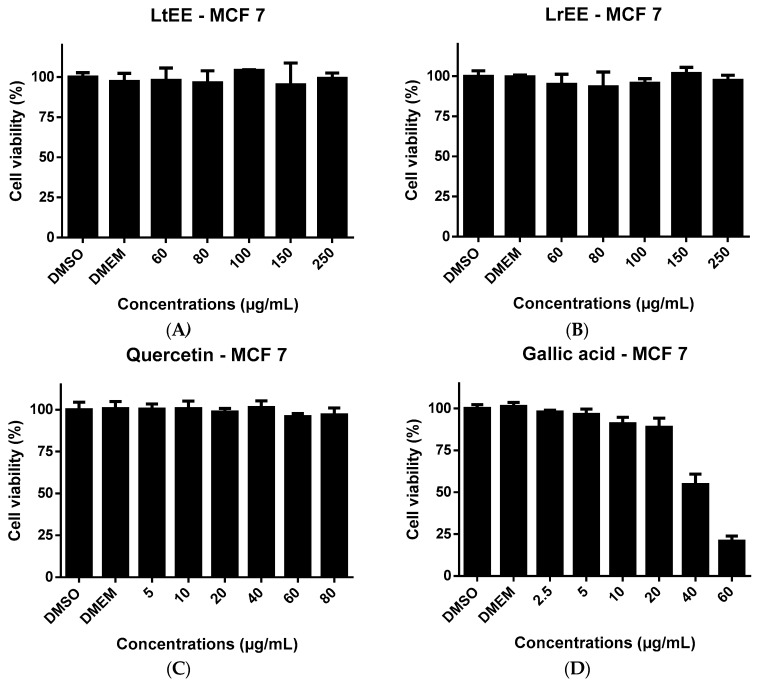
Cell viability of the human breast adenocarcinoma cell line MCF-7 upon 24 h exposure to ethanolic seeds extracts of *Licania tomentosa* (**A**); *L. rigida* (**B**); quercetin (**C**) and gallic acid (**D**). DMSO, dimethyl sulfoxide; DMEM: Dulbecco's Modified Eagle's Medium; LrEE: *L. rigida* ethanolic extract; LtEE: *L. tomentosa* ethanolic extract.

**Figure 4:**
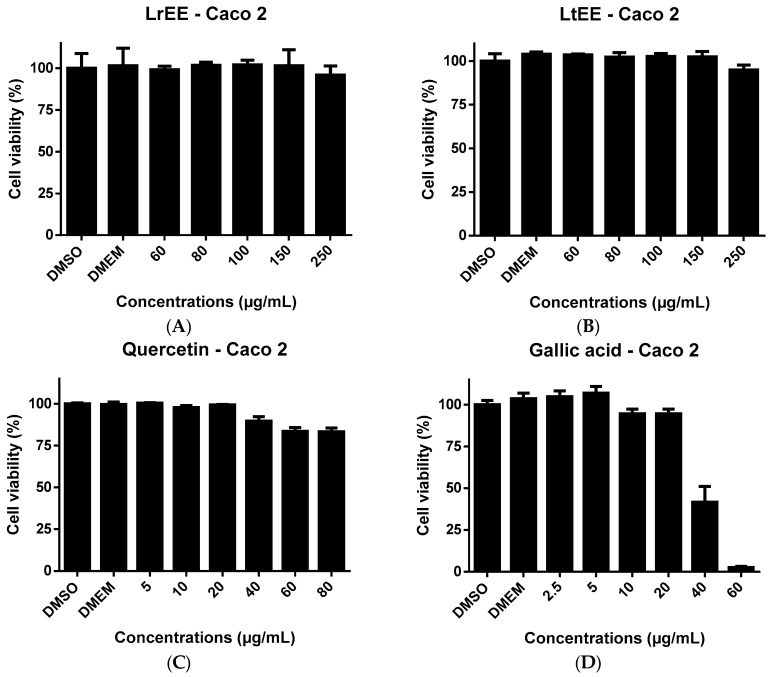
Cell viability of the human colon adenocarcinoma cell line Caco-2 upon 24 h exposure to ethanolic seeds extracts of *Licania rigida* (**A**); *L. tomentosa* (**B**); quercetin (**C**) and gallic acid (**D**). DMSO, dimethyl sulfoxide; DMEM, Dulbecco's Modified Eagle's Medium; LrEE, *L. rigida* ethanolic extract; LtEE, *L. tomentosa* ethanolic extract.

Since several studies have demonstrated the cytotoxic effects of flavonoids [36,37,38] and phenolic acids [39,40] to human cancer cells, in the present study LrEE and LtEE were tested for possible cytotoxicity towards the human MCF-7 breast cancer and Caco-2 colorectal adenocarcinoma cell lines. Also the flavonoid quercetin and phenolic acid galic acid, which both are constituents of the ethanolic extracts, were included in the cytotoxicity experiments for comparison. After treatment of both cell lines for 24 h with increasing concentrations of LrEE, LtEE, quercetin and gallic acid, cell viability was determined using the ATPlite assay. It was not possible to test the ethanol extracts at concentrations exceeding 250 µg/mL, due to the formation of precipitates in the culture medium. The same was true for quercetin at concentrations higher than 80 µg/mL medium. Flavonoids are known to easily precipitate in aqueous medium [41]. LrEE and LtEE concentrations up to 250 µg/mL culture medium did not show any toxicity to MCF-7 cells (Figure 3) and Caco-2 cells (Figure 4). The higher concentrations of quercetin were slightly cytotoxic to Caco-2 (Figure 4) but not to MCF-7 (Figure 3).

Gallic acid at 40 and 60 µg/mL resulted in clear cytotoxicity to both cell lines. The lack of cytotoxicity of LrEE and LtEE is in contrast with previous finding by ohers. Extracts of several plants from the Licania genius have been reported to exert in vitro cytotoxic effects towards cancer cell lines, for example extracts of L. heteromorpha [42] and L. michauxii in human hepatoma HepG2 cells and Caco-2 cells [43]. This discrepancy might be related to differences in extract preparation and type of viability assay used, e.g., ATPlite versus MTT assays.

## 3. Materials and Methods

### 3.1. Plant Material

Fruits from *L. rigida* and *L. tomentosa* were harvested (at least 3 kg each) in January, 2013, within an area of 0.384 km² in the Pici Campus at Federal University of Ceará (3°43’54.7032’’ S, 38°31’36.0084’’ W), in the city of Fortaleza, Northeastern Brazil. Plants were identified by the taxonomist Dr. Edson de Paula Nunes and voucher specimens of *L. rigida* and *L. tomentosa* (EAC 40216 and EAC 40215, respectively) were deposited at Herbarium Prisco Bezerra (EAC) at the same University. The seeds from freshly harvested plant material were separated from other plant materials, air-dried, and ground in a laboratory mill to a fine powder (particle size < 0.5 mm).

### 3.2. Chemicals

Gallic acid, chlorogenic acid, ellagic acid, ascorbic acid, ferrous sulfate, tannic acid, phenantroline and caffeic acid were purchased from Merck (Darmstadt, Germany). 2,2-Diphenyl-1-picrylhydrazyl (DPPH), thiobarbituric acid (TBA), 1,1,3,3-tetraethoxypropane (MDA), bovine serum albumin (BSA), agarose type I, sodium azide, aluminium chloride, potassium acetate, quercetin, quercitrin, isoquercitrin, rutin, kaempferol, catechin, epicatechin and Folin–Ciocalteu’s phenolic reagent were acquired from Sigma Aldrich Co. (St. Louis, MO, USA). All other chemicals used were of analytical grade.

### 3.3. Extract Preparation and Fractioning

The grounded seeds were submitted to extended extraction for 9 days with solvent (95% ethanol) being changed every 72 h (1:2, *w*/*v*) at room temperature, them the extracts were filtered and distilled in the rotary evaporator (40–50 °C), under low pressure to obtain the LtEE and LrEE [44]. Part of these extracts were used for fractioning described as follows. First, 25 g of each ethanolic seed extract were mixed with silica until the obtainment of a homogeneous mixture. Then, using paper filter and Büchner funnels, the mixture was eluted with solvents in crescent polarity (hexane, chloroform, ethyl acetate, methanol and water). This process was repeated until the eluate of each solvent used was totally transparent. Finally, the solvents were evaporated in the rotary evaporator (in the same conditions used to ethanolic extract), producing five fractions from *L. tomentosa* (LtHF, *L. tomentosa* hexanic fraction; LtCF, *L. tomentosa* chloroformic fraction; LtEAF, *L. tomentosa* ethyl acetate fraction; LtMF, *L. tomentosa* methanolic fraction; LtAF, *L. tomentosa* aqueous fraction) and other five from *L. rigida* (LrHF*, L. rigida* hexanic fraction; LrCF, *L. rigida* chloroformic fraction; LrEAF, *L. rigida* ethyl acetate fraction; LrMF, *L. rigida* methanolic fraction; LrAF, *L. rigida* aqueous fraction). The hexanic and chloroformic fractions were discarded because of insignificant antioxidant activity (previously detected). The extracts and fractions were stored under −4 °C until use for the analyses.

### 3.4. Chemical Characterization

#### 3.4.1. Phytochemical Screening

LtEE and LrEE were subjected to preliminary phytochemical screening for the detection of major classes of secondary metabolites by chemical reactions that result in the development of color and/or precipitate distinctive for each class of substances. Residues from both extracts were resuspended with suitable solvents for testing phenols and tannins (reaction with ferric chloride); saponins (foam test); triterpenoids and steroids (Liberman-Buchard test); different subclasses of flavonoids (tests of pH variation with sodium hydroxide and sulfuric acid) and alkaloids (reaction with Dragendorff reagent) [44].

#### 3.4.2. Total Phenolic Compounds Determination

The total phenol content of *L. tomentosa* and *L. rigida* extracts and respective fractions was determined by the Folin-Ciocalteu method [45], with some modifications. In a 96-well microplate, 50 µL of the samples were mixed with 50 µL of Folin-Ciocalteu’s reagent (33% *v*/*v*) and after 3 min, 100 µL of distilled water and 100 µL of sodium carbonate (7.5%) were, then, added. The reaction mixture was incubated for 30 min in the dark and the absorbance was measured at 700 nm in a spectrophotometer (Epoch, Biotek Intruments Inc., Winooski, VT, USA). Gallic acid (GA) was used as standard for phenolic compounds. The average of the readings was used and the total phenolic content was expressed as mg of gallic acid equivalent pergram of sample. Traditionally, gallic acid is used as standard [31]. All samples were processed in triplicates.

#### 3.4.3. Total Flavonoid Content Determination

The aluminum chloride colorimetric method was used to determine flavonoids content in the samples. Traditionally, quercetin is used to make the calibration curve [46]. Briefly, 10 mg of quercetin was dissolved in 80% ethanol, this stock solution was diluted to concentrations of 25, 50 and 100 µg/mL. The diluted standard solutions (0.5 mL) were separately mixed with 1.5 mL of 95% ethanol, 0.1 mL of 10% aluminum chloride, 0.1 mL of 1 M potassium acetate and 2.8 mL of distilled water. After incubation at room temperature for 30 min, the absorbance of the reaction mixture was measured at 415 nm using a spectrophotometer (Biospectro SP-220, Curitiba, Brasil). The amount of 10% aluminum chloride was substituted by the same amount of distilled water as a blank. Similarly, 0.5 mL of ethanol extracts were reacted with aluminum chloride for determination of flavonoid content as described above. The results were expressed as mg of quercetin equivalent per gram of sample. All samples were processed in triplicates.

#### 3.4.4. Tannin by Radial Diffusion Method

The determination of tannins of the ethanolic extract and the ethyl acetate, metanolic and aqueous fractions was performed by the radial diffusion method [47]. This method consists in the reaction between tannins and proteins in agarose gel forming a visible and measurable ring. First, a buffer of 50 mM acetic acid, 60 μM ascorbic acid and 0.04% sodium azide was prepared and adjusted to pH 5.0. This buffer was then used to prepare the gel by adding 1% agarose. Briefly, the solution was heated until complete agarose homogenization and, then, it was cooled down to 45 °C. At this point, 0.1% BSA was added. Quickly, the gel was distributed in aliquots of 15 mL in 9.0 cm in diameter Petri plates, placed into cooling to form gel for 10 min under refrigeration. Four-millimeter diameter wells were made on the gel 2.0 cm apart from each other and from the plate edges. By using a micropipette, four successive aliquots of 10 μL of each extract were added inside the wells. To obtain the calibration curve, the same protocol was performed along with serial dilutions made out from an ethanolic solution (70%) of tannic acid as a standard (proposed by the method) at a concentration of 25 mg/mL. All samples were processed in triplicates and the results were expressed as mg of tannic acid equivalent per gram of the sample.

#### 3.4.5. HPLC-DAD

##### Apparatus

High performance liquid chromatography (HPLC-DAD) was performed with a Shimadzu Prominence Auto Sampler (SIL-20A) HPLC system (Shimadzu, Kyoto, Japan), equipped with Shimadzu LC-20AT reciprocating pumps connected to a DGU 20A5 degasser with a CBM 20A integrator, SPD-M20A diode array detector and LC solution 1.22 SP1 software (Shimadzu, Kyoto, Japan).

##### Procedures

Reverse phase chromatographic analyses were carried out under gradient conditions using a C_18_ column (4.6 mm × 250 mm) packed with 5 μm diameter particles; the mobile phase consisted of water containing 2% acetic acid (A) and methanol (B), and the composition gradient was: 5% (B) for 2 min; 25% (B) until 10 min; 40%, 50%, 60%, 70% and 80% (B) every 10 min; following the method [48] with slight modifications. The samples of *L. tomentosa* and *L. rigida* (ethanolic extract, and methanolic, ethyl acetate, chloroform and aqueous fractions) and mobile phase were filtered through 0.45 μm membrane filter (Millipore, Darmstadt, Germany) and then degassed by ultrasonic bath prior to use, the samples were analyzed at a concentration of 20 mg/mL. The flow rate was 0.7 mL/min and the injection volume was 40 μL. The sample and mobile phase were filtered through 0.45 μm membrane filter (Millipore) and then degassed by ultrasonic bath prior to use. Stock solutions of standard references were prepared in the HPLC mobile phase at a concentration range of 0.030–0.250 mg/mL catechin, epicatechin, quercetin, quercitrin, isoquercitrin, kaempferol and rutin, and 0.045–0.500 mg/mL for gallic, chlorogenic, ellagic and caffeic acids. Quantification was carried out by integration of the peaks using the external standard method, at 257 nm for gallic acid, 280 nm for catechin and epicatechin, 325 nm for chlorogenic, ellagic and caffeic acids, and 365 for quercetin, quercitrin, isoquercitrin, rutin kaempferol and kaempferol glycoside. The results were expressed as mg of each compound per gram of sample. The chromatography peaks were confirmed by comparing its retention time with those of reference standards and by DAD spectra (200 to 600 nm). All chromatography operations were carried out at room temperature and in triplicate. LOD and LOQ (“Limit of Detection” and “Limit of Quantification”) were calculated based on the standard deviation of the responses and the slope using three independent analytical curves [49]. LOD and LOQ were calculated as 3.3 and 10 σ/S, respectively, where σ is the standard deviation of the response and S is the slope of the calibration curve.

### 3.5. In Vitro Assessment of Antioxidant Activity

#### 3.5.1. DPPH Radical Scavenging

The antioxidant activity was evaluated using the in vitro photocolorimetric method based on the DPPH scavenging capacity [50]. Shortly, it was prepared a solution of 60 µmol/L DPPH in ethanol. In a 96-well microplate, 100 µL of DPPH solution was added to 100 µL of the test sample dissolved in ethanol at different concentrations ranging from 0.5 to 512 μg/mL, in nine replicates. As negative control, it was used 100 μL DPPH solution and 100 μL of ethanol. The reaction was incubated in the dark at room temperature for 30 min and the readings were executed in a spectrophotometer at 518 nm (Biotek Instruments Inc. Epoch, Winooski, VT, USA). The DPPH-scavenging activity was calculated by the following formula:

%SA = 100 × (Abs_control_ − Abs_sample_)/Abs_control_ (1)

where SA is the scavenging activity; Abs_control_ is the absorbance of control; and Abs_sample_ is the absorbance of the samples. The concentration of the sample needed to scavenge 50% of DPPH radical (SC_50_), was obtained by logistic regression plotting the DPPH-scavenging percentage of each sample against the logarithm of sample concentration. Ascorbic acid was used as positive control in this experiment at the same concentrations used for the samples. All samples were processed in triplicates.

#### 3.5.2. TBARS Assay

Due to the fact Reactive Oxygen Species (ROS) have an extremely short half-life, they are difficults to measure directly. Instead, several products of the damage produced by oxidative stress can be measured such as thiobarbituric acid reactive substances (TBARS). These substances are formed as a byproduct of lipid peroxidation, which can be detected using thiobarbituric acid as a reagent. MDA is one of several low-molecular-weight final products formed via the decomposition of lipid peroxidation products. The TBARS formation was determined using egg yolk phospholipids [51]. Concisely, the phospholipids, obtained from homogenate of egg yolk membrane, were added to the reaction mixture containing various concentrations of the extracts (LrEE and LtEE) and their respective ethyl acetate, methanol and aqueous fractions (8 to 250 µg/mL) with deionized water to complement the total volume of 500 µL. Then, the reaction mixture was pre-incubated at 37 °C for 1 h in the presence or absence of iron (75 µM) as a lipid peroxidation stress inducer. The reaction’s color was observed by adding 500 µL of acetate buffer (20%) and 500 µL of TBA (0.6%) water solution and, then, incubated at 97 °C in water bath for 1 h. In the meantime, a series of dilutions of 0.03 mM MDA standard were incubated in the same manner. The absorbance was measured at wavelength of 532 nm (Biospectro SP-220). All samples were processed in triplicates. The concentration of the sample needed to cause 50% inhibition of lipid peroxidation (IC_50_) was obtained by logistic regression plotting the lipid peroxidation per cent inhibition of each sample against the logarithm of sample concentration.

#### 3.5.3. Iron chelating Activity

To examine the iron chelating properties of the extracts (LrEE and LtEE) and their respective ethyl acetate, methanol and aqueous fractions, it was used the *o*-phenanthroline method with slight modifications [52]. Briefly, the mixture, containing 40 µL of Fe^2+^ (120 µM Fe_2_SO_4_) and 20 µL of the samples at concentrations from 62.5 to 1000 µg/mL, was added to 591 µL of water plus 376 µL of Tris-HCl buffer (0.1 M; pH 7.2), followed by 5 min wait to form complex(es) between Fe^2+^ and the related compounds. After that, 13 µL of *o*-phenanthroline (0.25%) solution was added to determine the colored complex(es) formation between *o*-phenanthroline and free Fe^2+^. The absorbance was recorded at 510 nm (Biospectro SP-220). The values are expressed in percentage of control determined in the absence of the samples. Solutions of Fe_2_SO_4_ were made in distilled water just before use. EDTA (Ethylenediamine tetraacetic acid) was used as positive control for its acknowledged chelating capacity, in the same concentration of the samples. All samples were processed in triplicate.

### 3.6. Cell Culture and Cell Viability Assay

The human MCF-7 breast cancer and Caco-2 colorectal adenocarcinoma cell lines were purchased from ATCC (Manassas, VA, USA). Cells were routinely grown in Dulbecco’s Modified Eagle’s Medium (DMEM, Lonza, Verviers, Belgium), supplemented with 10% (*v*/*v*) fetal calf serum (FCS, Gibco, Carlsbad, CA, USA), 0.6% (*v*/*v*) penicillin-streptomycin (10,000 units penicillin and streptomycin 10 mg/mL (Sigma-Aldrich Co.), and 1% (*v*/*v*) non-essential amino acids (NEAA, Lonza, Verviers, Belgium) at 37 °C in a humidified atmosphere containing 5% CO_2_.

MCF-7 cells and Caco-2 cells were cultured to 80% confluency, trypsinized, counted, and resuspended in culture medium to a final cell suspension density of 100,000 cells/mL. Afterwards, 100 µL of cell suspension were seeded into each well of 96-well flat bottom microtiter plates. Upon culturing cells for 48 h, cells were exposed for 24 h to extracts and compounds at final concentrations rangingfrom 60–250 µg/mL for LrEE and LtEE; 5–80 µg/mL for quercetin and 2.5–60 µg/mL for gallic acid. The highest concentrations were chosen based on characteristics of each sample, i.e., larger amounts of the extracts and quercetin induced precipitation in the culture medium, and caffeic acid and gallic acid changed the pH of the medium. DMSO, added to the medium at a final concentration of 0.05% (*v*/*v*), was used as the solvent control.

The ATPlite assay (PerkinElmer, Groningen, The Netherlands) was performed to evaluate cell viability according to the manufacturer’s instructions. After 24 h exposure of the cells to the extracts and compounds, the culture medium (100 µL) in each well was refreshed, 50 µL of the mammalian cell lysis solution (ATPlite kit) was added and the plate was shaken for 2 min using a microtiter plate shaker. Then, 50 µL of substrate solution (ATPlite kit) was added to each well followed by shaking for 10 min. Thereafter, the luminescence was measured using a multi-mode microplate reader (BioTek Synergy HT). Cell viability was calculated using the equation: luminescence_treated_/luminescence_solventcontrol_ × 100%. The assay was performed in triplicate. A cytotoxic concentration is a concentration resulting in <80%–100% viability [53,54].

### 3.7. Statistical Analysis

The results are given as means ± standard deviation (SD). Student’s *t*-test was used for comparison between two means and one-way analysis of variance (ANOVA) was used for comparison of more than two means. A difference was considered statistically significant when *p* < 0.05. The excessively high degree of colinearity among the chemical compounds concentration hindered the use of regression analysis, even resorting to orthogonalization techniques such as principal components analysis. Strong collinearity among the independent variables of a regressions leads either to imprecise estimates of the coefficients, or even to failure to determine them in a unique manner. In this case collinearity was of the second type. Thus, to define the effects of the chemical compounds on antioxidant activity, partial correlation analysis, which isolates the effect of other variables on the pair of variables being analyzed, was performed [55].

## 4. Conclusions

The seed extracts of *L. tomentosa* and *L. rigida* showed promising phenolic profiles with high concentrations of compounds having antioxidant activity, but no cytotoxicity towards the tested cancer cell lines. When considering the scavenging of free radicals, flavonoids compounds such as quercetin, quercitrin and catechin were the preponderant antioxidant molecules, whereas phenolic acids showed greater importance when stressed conditions were used (presence of iron). The compounds may be exploited in a near future in the pharmaceutical or nutraceutical industries and this consequently extendeds their well-known use as folk remedies, adding value to these wild plants. Although there are no reports on any hazards based on the history of use of these fruit seeds, further toxicological and antioxidant in vivo tests must be run in order to guarantee their safe use and efficacy of these species in the combat against oxidation in biological systems.

## Figures and Tables

**Table 1:** Phytochemical screening of chemical compound classes in the ethanolic extracts of *Licania rigida* and *L. tomentosa* seeds.

Chemical Compound Classes	Extract	
	LrEE	LtEE
Hydrolysable tannins	+	+
Anthocyanins/anthocyanidins	−	−
Flavonols/xanthones	+	+
Chalcones/auronas	−	−
Flavononols	+	+
Leucoanthocyanidins	−	−
Flavones	+	+
Catechins	+	+
Steroids/triterpenoids	−	−
Flavonones	+	+
Saponins	+	+

+, −: indicates presence and absence, respectively; LrEE: *L. rigida* ethanolic extract; LtEE: *L. tomentosa* ethanolic extract.

**Table 2:** Total phenolics, flavonoid and tannin content of *Licania tomentosa* and *L. rigida* seed extracts.

Samples	Chemical Characterization		
	Total Phenolics *	Flavonoids **	Tannins ***
LtEE	108.47 ± 6.90 ^a^	10.91 ± 2.24 ^a^	0.059 ± 0.017 ^a^
LtMF	96.95 ± 0.51 ^a^	35.56 ± 1.88 ^b^	0.039 ± 0.017 ^a^
LtEAF	201.83 ± 4.27 ^c^	90.61 ± 8.87 ^c^	-
LtAF	111.15 ± 0.64 ^a^	ND	0.182 ± 0.007 ^b^
LrEE	206.98 ± 5.18 ^c^	26.94 ± 1.13 ^b^	0.147 ± 0.011 ^b^
LrMF	200.52 ± 2.93 ^c^	14.57 ± 0.29 ^a^	0.159 ± 0.037 ^b^
LrEAF	166.95 ± 3.58 ^b^	ND	-
LrAF	119.21 ± 1.15 ^a^	1.91 ± 0.19 ^d^	0.294 ± 0.027 ^c^

LtEE: *L. tomentosa* ethanolic extract; LtMF: *L. tomentosa* methanolic fraction; LtEAF: *L. tomentosa* ethyl acetate fraction; LtAF: *L. tomentosa* aqueous fraction; LrEE: *L. rigida* ethanolic extract; LrMF: *L. rigida* methanolic fraction; LrEAF: *L. rigida* ethyl acetate fraction; LrAF: *L. rigida* aqueous fraction. ND: not determined. -: not detected. Values are means ± SEM of three measurements. Similar superscript letters in the same column do not differ significantly (*p* > 0.05; ANOVA). * Gallic acid equivalent in mg per g of the sample. ** Quercetin equivalent in mg per g of the sample. *** Tannic acid equivalent in mg per g of the sample.

**Table 3:** Phenolics composition of *Licania rigida* by HPLC-DAD analysis.

Compounds	LrEE		LrMF		LrEAF		LrCF		LrAF		Calibration Curve	RT	LOQ	LOD
	mg/g	%	mg/g	%	mg/g	%	mg/g	%	mg/g	%		min	μg/mL	μg/mL
Gallic acid	3.41 ± 0.02 ^a^	0.34	5.98 ± 0.01 ^a^	0.59	8.05 ± 0.01 ^a^	0.80	2.81 ± 0.03 ^a^	0.28	1.57 ± 0.01 ^a^	0.15	*Y* = 14,063*x* + 1187.9 (r = 0.9997)	12.53	0.018	0.059
Catechin	1.57 ± 0.01 ^b^	0.15	3.15 ± 0.03 ^b^	0.31	7.84 ± 0.02 ^a^	0.78	1.35 ± 0.02 ^b^	0.13	2.74 ± 0.01 ^b^	0.27	*Y* = 11,964*x* + 1387.6 (r = 0.9999)	15.96	0.023	0.075
Chlorogenic acid	15.68 ± 0.01 ^c^	1.56	14.08 ± 0.02 ^c^	1.40	39.25 ± 0.02 ^b^	3.92	17.46 ± 0.01 ^c^	1.74	1.61 ± 0.02 ^a^	0.16	*Y* = 12,850*x* + 1372.5 (r = 0.9993)	21.09	0.007	0.023
Caffeic acid	13.95 ± 0.01 ^d^	1.39	16.19 ± 0.01 ^d^	1.61	31.19 ± 0.01 ^c^	3.11	6.95 ± 0.01 ^d^	0.69	4.83 ± 0.03 ^c^	0.48	*Y* = 12,748*x* + 1240.8 (r = 0.9991)	25.01	0.034	0.112
Epicatechin	1.30 ± 0.02 ^b^	0.13	2.73 ± 0.01 ^b^	0.27	14.27 ± 0.03 ^d^	1.42	3.11 ± 0.01 ^a^	0.31	0.92 ± 0.02 ^d^	0.09	*Y* = 12,678*x* + 1329.7 (r = 0.9990)	30.76	0.015	0.049
Rutin	2.97 ± 0.03 ^a^	0.29	7.39 ± 0.03 ^e^	0.73	14.63 ± 0.01 ^d^	1.46	-	-	2.95 ± 0.02 ^b^	0.29	*Y* = 12,756*x* + 1367.1 (r = 0.9996)	41.15	0.029	0.095
Quercitrin	1.25 ± 0.01 ^b^	0.12	4.52 ± 0.01 ^f^	0.45	38.94 ± 0.01 ^b^	3.89	4.62 ± 0.01 ^e^	0.46	1.68 ± 0.03 ^a^	0.16	*Y* = 12,694*x* + 1357.4 (r = 0.9993)	44.23	0.042	0.138
Isoquercitrin	6.13 ± 0.01 ^e^	0.61	7.06 ± 0.02 ^e^	0.70	13.85 ± 0.02 ^d^	1.38	-	-	2.53 ± 0.01 ^b^	0.25	*Y* = 12,571*x* + 1358.5 (r = 0.9997)	46.37	0.031	0.102
Quercetin	3.28 ± 0.02 ^a^	0.32	8.91 ± 0.01 ^f^	0.89	47.02± 0.01 ^e^	4.70	-	-	8.37 ± 0.01 ^e^	0.83	*Y* = 14,274*x* + 1341.5 (r = 0.9995)	50.02	0.008	0.029
Kaempferol	12.65 ± 0.01 ^f^	1.26	14.57 ± 0.02 ^c^	1.34	38.61 ± 0.01 ^b^	3.86	-	-	4.19 ± 0.02 ^f^	0.41	*Y* = 13,657*x* + 1293.8 (r = 0.9999)	55.19	0.021	0.069
Kaempferol glycoside	5.49 ± 0.01 ^e^	0.54	3.38 ± 0.01 ^b^	0.33	14.95 ± 0.01 ^d^	1.49	-	-	1.07 ± 0.01 ^d^	0.10	-	60.27	-	-

Results are expressed as mean ± S.E. of three determinations. Concentrations are expressed as mg of each compound per g of sample and in % (g of each compound per 100 g of sample). LrEE: *Licania rigida* ethanolic extract; LrMF: *L. rigida* methanolic fraction; LrEAF: *L. rigida* ethyl acetate fraction; LrAF, LrCF: *L. rigida* chloroformic fraction and *L. rigida* aqueous fraction; LOD: Limit of detection; LOQ: limit of quantification; RT: retention time. Values with different letters differ by Tukey test at *p* < 0.05.

**Table 4:** Phenolics composition of *Licania tomentosa* by HPLC-DAD analysis.

Compounds	LtEE		LtMF		LtEAF		LtCF		LtAF		Calibration Curve	RT	LOQ	LOD
	mg/g	%	mg/g	%	mg/g	%	mg/g	%	mg/g	%		min	μg/mL	μg/mL
Gallic acid	5.17 ± 0.03 ^a^	0.51	6.81 ± 0.01 ^a^	0.68	24.35 ± 0.01 ^a^	2.43	3.67 ± 0.01 ^a^	0.36	6.93 ± 0.02 ^a^	0.69	*Y* = 14,063*x* + 1187.9 (r = 0.9997)	12.53	0.018	0.059
Catechin	1.43 ± 0.02 ^b^	0.14	1.54 ± 0.01 ^b^	0.15	7.81 ± 0.03 ^b^	0.78	3.51 ± 0.02 ^a^	0.35	1.34 ± 0.01 ^b^	0.13	*Y* = 11,964*x* + 1387.6 (r = 0.9999)	15.96	0.023	0.075
Chlorogenic acid	22.38 ± 0.01 ^c^	2.23	14.63 ± 0.02 ^c^	1.46	46.59 ± 0.02 ^c^	4.65	14.38 ± 0.02 ^b^	1.43	3.81 ± 0.02 ^c^	0.38	*Y* = 12,850*x* + 1372.5 (r = 0.9993)	21.09	0.007	0.023
Caffeic acid	14.90 ± 0.01 ^d^	1.49	5.57 ± 0.03 ^d^	0.55	42.63 ± 0.01 ^d^	4.26	3.82 ± 0.02 ^a^	0.33	2.67 ± 0.03 ^d^	0.26	*Y* = 12,748*x* + 1240.8 (r = 0.9991)	25.01	0.034	0.112
Epicatechin	3.25 ± 0.03 ^e^	0.32	1.30 ± 0.01 ^b^	0.13	9.21 ± 0.03 ^b^	0.92	0.59 ± 0.01 ^c^	0.05	2.72 ± 0.02 ^d^	0.27	*Y* = 12,678*x* + 1329.7 (r = 0.9990)	31.87	0.015	0.049
Ellagic acid	4.61 ± 0.04 ^a^	0.46	2.14 ± 0.02 ^b^	0.21	31.98 ± 0.01 ^e^	3.19	3.16 ± 0.03 ^a^	0.31	6.83 ± 0.01 ^a^	0.68	*Y* = 12,756*x* + 1367.1 (r = 0.9996)	37.26	0.011	0.034
Rutin	8.73 ± 0.01 ^f^	0.87	14.09 ± 0.03 ^c^	1.40	20.14 ± 0.01 ^f^	2.01	-	-	7.40 ± 0.02 ^a^	0.74	*Y* = 12,694*x* + 1357.4 (r = 0.9993)	43.15	0.029	0.095
Quercitrin	3.56 ± 0.01 ^e^	0.35	5.32 ± 0.01 ^d^	0.53	24.17 ± 0.04 ^a^	2.41	0.24 ± 0.01 ^c^	0.02	5.01 ± 0.03 ^e^	0.50	*Y* = 12,571*x* + 1358.5 (r = 0.9997)	45.16	0.042	0.138
Quercetin	14.27 ± 0.02 ^d^	1.42	9.76 ± 0.01 ^e^	0.97	17.54 ± 0.01 ^a^	2.75	-	-	14.27 ± 0.01 ^f^	1.42	*Y* = 14,274*x* + 1341.5 (r = 0.9995)	50.02	0.008	0.029
Kaempferol	4.83 ± 0.01 ^a^	0.48	5.41 ± 0.02 ^d^	0.54	2.68 ± 0.01 ^g^	0.26	3.47 ± 0.02 ^a^	0.34	0.89 ± 0.02 ^g^	0.08	*Y* = 13,657*x* + 1293.8 (r = 0.9999)	55.19	0.021	0.069
Kaempferol glycoside	1.56 ± 0.01 ^b^	0.15	1.70 ± 0.01 ^b^	0.17	8.39 ± 0.01 ^b^	0.83	0.73 ± 0.01 ^c^	0.07	3.15 ± 0.01 ^d^	0.31	-	59.83	-	-

Results are expressed as means ± S.E. of three determinations. Concentrations are expressed as mg of each compound per g of sample and in % (g of each compound per 100 g of sample). LtMF: *L. tomentosa* methanolic fraction; LtEAF: *L. tomentosa* ethyl acetate fraction; LtCF: *L. tomentosa* chloroformic fraction and LtAF: *L. tomentosa* aqueous fraction; LOD: Limit of detection; LOQ: limit of quantification; RT: retention time. Values with different letters differ by Tukey test with *p* < 0.05.

**Table 5:** Antioxidant activities of *Licania tomentosa* and *L. rigida* seed extracts and fractions. The scavenging activities were expressed as 50% scavenging concentration (SC_50_) for the DPPH assay, and as 50% inhibiting concentration (IC_50_) for the TBARS assay.

Samples	DPPH Assay SC_50_ (µg/mL)	TBARS Assay IC_50_ (µg/mL)	
		Without Iron Stress	With Iron Stress
LtEE	27.14 ± 1.96 ^b^	236.07 ± 23.76 ^d^	20.71 ± 2.89 ^b^
LtMF	26.30 ± 1.86 ^b^	105.72 ± 32.53 ^c^	8.55 ± 3.97 ^a^
LtEAF	248.8 ± 2.95 ^f^	86.57 ± 15.97 ^b^	1,163.33 ± 115.1 ^e^
LtAF	42.68 ± 1.22 ^c^	ND	ND
LrEE	174.63 ± 11.40 ^e^	144.33 ± 20.36 ^c^	286.57 ± 15.05 ^d^
LrMF	84.70 ± 4.30 ^d^	445.17 ± 46.80 ^e^	ND
LrEAF	9.15 ± 0.03 ^a^	ND	244.17 ± 67.15 ^d^
LrAF	38.00 ± 0.883 ^c^	60.80 ± 8.94 ^a^	153.13 ± 17.65 ^c^
Positive control			
Ascorbic Acid	10.17 ± 0.74 ^a^		

LtEE: *L. tomentosa* ethanolic extract; LtMF: *L. tomentosa* methanolic fraction; LtEAF: *L. tomentosa* ethyl acetate fraction; LtAF: *L. tomentosa* aqueous fraction; LrEE: *L. rigida* ethanolic extract; LrMF: *L. rigida* methanolic fraction; LrEAF: *L. rigida* ethyl acetate fraction; LrAF: *L. rigida* aqueous fraction. ND: not determined. ^a−f^ Values with different letters at the same column differ significantly (*p* < 0.05).

**Table 6:** Partial correlation coefficients between phenolic compounds composition (HPLC) versus antioxidant activities.

Phenolic Compounds	Antioxidant Activities		
	DPPH	TBARS Without Iron	TBARS With Iron
Phenolic acids	−0.410	−0.371	0.246
Caffeic acid	−0.654	0.023	−0.117
Chlorogenic acid	−0.268	−0.552	0.465
Ellagic acid	0.112	0.013	−0.074
Gallic acid	0.001	−0.154	0.180
Flavonoids	0.410	0.371	−0.246
Catechin	0.109	0.690	−0.664
Epicatechin	0.517	−0.068	0.033
Isoquercitrin	−0.520	0.262	−0.226
Kaempferol	−0.415	0.002	0.027
Kaempferol glucoside	−0.546	0.235	−0.256
Quercetin	0.788	0.514	−0.336
Quercitrin	0.745	−0.214	0.043
Rutin	0.436	−0.265	0.346
